# Two sides of the same coin: recruitment performance and perceived workload in primary care trials-insights from the AgeWell.de study

**DOI:** 10.1186/s12875-025-02948-1

**Published:** 2025-08-05

**Authors:** Linda Sanftenberg, Robert Philipp Kosilek, Lorenz Birnberger, Hannah Schillok, Felix Wittmann, Melanie Luppa, Anne Blawert, Melanie Boekholt, Christian Brettschneider, Hans-Helmut König, Alexander Bauer, Solveig Weise, Thomas Frese, Hanna Kaduszkiewicz, Juliane Döhring, Catharina Escales, Jochen René Thyrian, Birgitt Wiese, Steffi G. Riedel-Heller, Jochen Gensichen

**Affiliations:** 1 Institute of General Practice and Family Medicine, University Hospital, LMU Munich, 80336 Munich, Germany; 2 Medical Faculty, Leipzig, Institute of Social Medicine, Occupational Health and Public Health (ISAP), 04103 Leipzig, Germany; 3 German Center for Neurodegenerative Diseases (DZNE), Site Rostock/Greifswald, 17489 Greifswald, Germany; 4 Department of Health Economics and Health Services Research, University Medical Center Hamburg-Eppendorf, 20246 Hamburg, Germany; 5 Institute of General Practice and Family Medicine, Martin-Luther-University Halle-Wittenberg, 06112 Halle, Germany; 6 Institute of General Practice, University of Kiel, 24105 Kiel, Germany; 7 Institute for Community Medicine, University Medicine Greifswald, 17487 Greifswald, Germany; 8 Work Group Medical Statistics and IT-Infrastructure, Institute for General Practice, Hannover Medical School, 30625 Hannover, Germany

**Keywords:** Patient recruitment, General practitioner, Barriers and facilitators, Randomized controlled trial, Practice-based research networks

## Abstract

**Background:**

Recruitment through general practitioners (GPs) is a key challenge in primary care trials. Understanding how individual, practice, and regional characteristics affect recruitment and perceived workload could help optimize participation strategies. This study aims to identify barriers and facilitators to patient recruitment within the AgeWell.de dementia prevention trial.

**Methods:**

We analysed publicly available data on GPs participating in the AgeWell.de trial, including their sociodemographic characteristics, practice structures, and socioeconomic indicators of their practice locations, alongside recruitment and survey data. We used correlation analysis and uni- and multivariable regression models to explore determinants of study engagement in terms of recruitment performance and perceived workload.

**Results:**

Among 120 participating GPs, a total of 1,173 patients were recruited, though contributions varied widely. The top 20% of recruiters (Q5) accounted for 42.1% of all participants, while the lowest quintile (Q1) recruited just 3.2%. GPs with a doctorate degree recruited more patients (IRR = 1.45, *p* < 0.05). Higher perceived workload was linked to increased recruitment engagement (IRR = 1.30, *p* < 0.1). In contrast, larger practice teams were associated with lower perceived workload (OR = 0.71, *p* < 0.1).

**Conclusion:**

GP recruitment performance and perceived workload are closely linked, influenced by both individual research interest and structural support. The disproportionate recruitment burden among a small subset of GPs highlights the need for strategies to engage low recruiters and support high performers. Strengthening practice-based research networks, could help make research involvement more feasible for a wider range of GPs.

*Trail registration*: German Clinical Trials Register (DRKS; trial identifier: DRKS00013555); Date of Registration: 2017-12-07.

**Supplementary Information:**

The online version contains supplementary material available at 10.1186/s12875-025-02948-1.

## Introduction

Strong primary care systems play a crucial role in delivering high-quality healthcare and ensuring equal health opportunities by providing comprehensive, accessible, and continuous care that considers social determinants of health [1, 2]. Conducting evidence-based research in primary care and integrating findings into routine practice is essential for improving patient outcomes [3, 4]. Randomized controlled trials (RCTs) are particularly important, as they provide the highest level of evidence for evaluating preventive, therapeutic, and rehabilitative interventions [5]. One such study is the AgeWell.de trial, a multicenter, cluster-randomized controlled study investigating a multicomponent intervention to prevent cognitive decline in older adults in German general practice [6, 7]. General practitioners (GPs) were eligible to study participation if they were already associated as a research practice of teaching practice with the respective study center. GPs had been invited to study participation by each study center using an invitation letter with information on study design and aims as well as GPs’ duties during the trial. GPs interested in study participation replied per email, fax or telephone. The recruiting study sites scheduled a personal appointment at the GP practice to explain the recruitment procedure and to provide all necessary study documents [6].

Recruiting patients for the AgeWell.de trial was challenging [8]. Since recruitment is a critical step in primary care research [9, 10], this analysis explores the barriers and facilitators of successful implementation. Study recruitment can be assessed through key factors such as reach, effectiveness, adoption, implementation, and maintenance [11]. In practice-based research networks (PBRNs), reach is particularly important, as it involves both the willingness of the GPs to participate and the support needed to recruit a representative sample. This includes clear communication, assessing structural conditions within GP practices, and identifying perceived barriers—both at the start of the study, during initial setup, and over time as recruitment progresses.

When implementing interventions, systematic and context-sensitive approaches can help to assess and address key factors, particularly in promoting health equity [12]. These approaches emphasize a multi-level perspective, considering partner characteristics, external influences, and infrastructure for long-term sustainability. They also encourage linking these structural factors to intervention components, allowing for tailoring and adaptation to improve implementation success. Ideally, such strategies should be integrated from the start of a clinical trial, but they can also provide valuable insights after implementation [13].

This analysis investigates factors influencing recruitment performance in the AgeWell.de trial, examining GPs’ perceived recruitment effort alongside their sociodemographic characteristics, practice structures, and the socioeconomic context of their practice locations.

## Methods

### Study subjects

This study utilizes data from the AgeWell.de trial, that has been conducted at five study sites in the regions of Leipzig, Kiel, Greifswald, Munich, and Halle (Germany). Altogether, 1,030 primary care patients have been included based on an elevated CAIDE risk score (Cardiovascular Risk Factors, Aging, and Incidence of Dementia) for dementia [14]. Participants randomized to the intervention group received a structured, multi-component lifestyle program targeting modifiable dementia risk factors over two years. The primary outcome was the delay in cognitive decline. A total of 123 GPs participated, primarily responsible for patient recruitment and cardiovascular risk optimization. For the control group, GPs were only responsible for identifying and recruiting patients. Full details on the trial’s design, intervention, and primary outcomes are available in the trial protocol and primary results publications [6, 7].

### Study design

This study focused on information regarding participating GPs, no patient data were analyzed. The number of patients recruited and the perceived workload during recruitment, personal and structural factors of GPs, their practices and the socioeconomic context were assessed. Personal factors that were evaluated referred to the age and gender of the respective GP, as well as his or her speciality in medical care and further qualifications (i.e., doctorate degree). Structural factors that were assessed referred to the individual GP practices and included the GP practice type (solo practice versus joint practice), the number of physicians per GP practice, the number of non-physician practice staff. Furthermore, characteristics of the areas where GP practices were located have been considered. Besides the number of inhabitants, the German Index of Social Deprivation (GISD score) and the GP coverage level of each area has been analysed.

The effectiveness of the implementation was analysed via correlation analysis and regression models using the above-mentioned structural factors as independent variables.

### Data collection and variables of interest

#### Publicly available data

Out of 123 participating GP practices in the AgeWell.de trial, 3 were excluded from this analysis because no sufficient data could be gathered. For the remaining 120 GPs, professional information about the individual GPs, their practices and the socio-economic context and equity parameters of the practice locations were evaluated. The data were collected from various publicly accessible sources, including information from practice websites, the respective Association of Statutory Health Insurance Physicians and from the Robert Koch Institute (RKI) [15–21]. The German Index of Social Deprivation (GISD score), which is based on income, education, and employment dimensions, measures the level of socioeconomic deprivation in a region, with a score of 1 indicating maximum deprivation and 0 indicating very low or no deprivation [22, 23].

#### Survey for GPs

Participating GPs were invited to answer a paper-based survey at the end of the two-year lifestyle intervention to assess the level of effort required to participate in the trial from their perspective. The survey included the collection of demographic data of physicians such as age (in years) and gender (male or female). The primary measure of interest was the overall perceived workload of participating in the trial, which was assessed on a 5-point Likert scale. The response options ranged from 1 ("Very low") to 5 ("Very high"). The questionnaire was developed for this study, an English language version is shown as supplementary file 1. The questionnaire was filled out by 66 out of 120 GPs, resulting in a response rate of 55%.

**Fig. 1:**
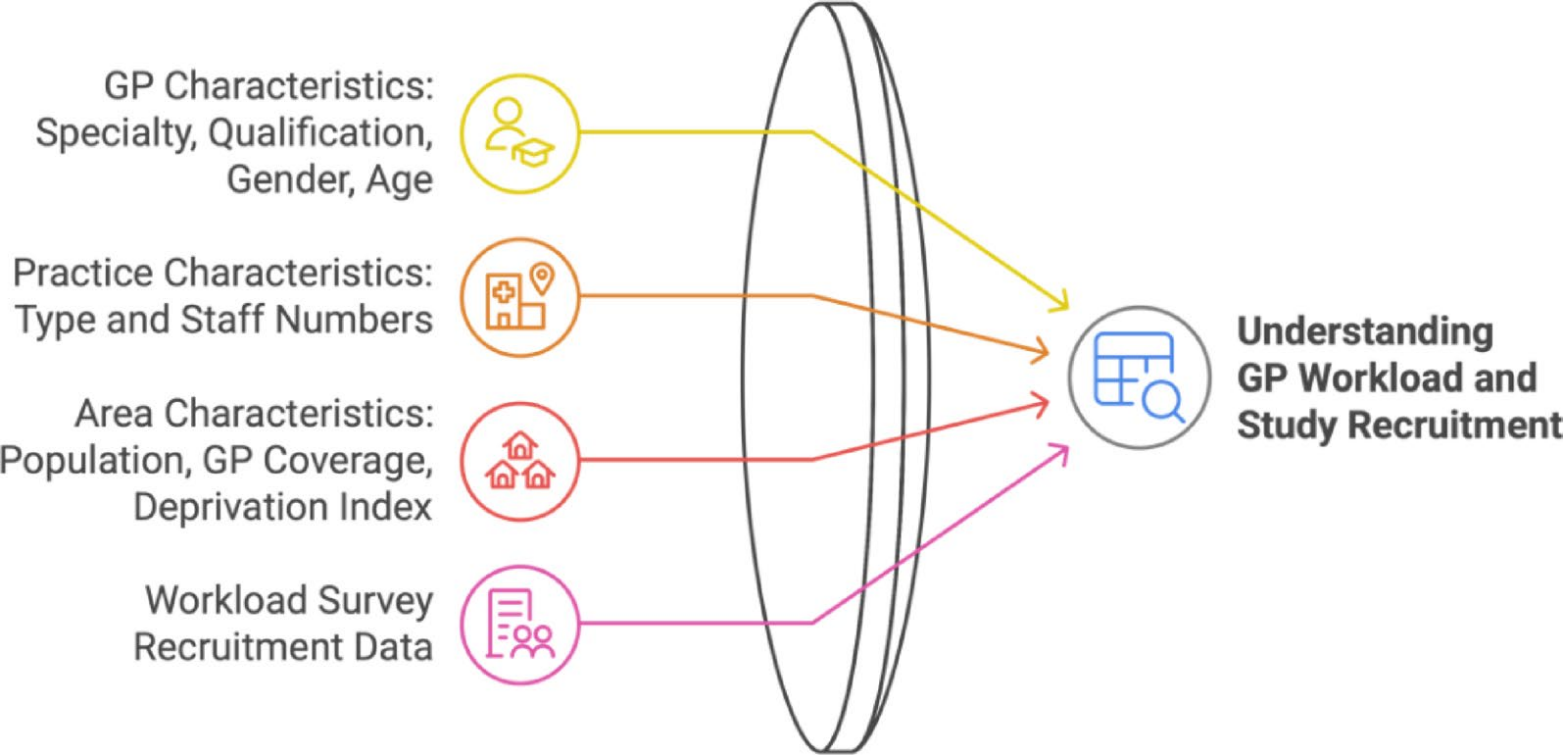
Conceptual framework for analyzing study workload and recruitment. Data on GP, practice, area and study characteristics were obtained from the AgeWell.de trial and public sources, including practice websites, the Association of Statutory Health Insurance Physicians, and the Robert Koch Institute. These components were combined to explore their relationship with GP participation and recruitment outcomes

The conceptual framework of the data collected and variables of interest is shown in Fig. 1, definitions and data sources are provided in supplementary file 2.

### Data analysis

We examined the influence of predictor variables including GP characteristics, practice structure and socioeconomic context on the two dependent variables patient recruitment and perceived recruitment workload.

For descriptive statistics, ordinal variables were reported as counts and percentages, and continuous variables as median and interquartile range.

To analyze recruitment patterns among GPs, we first categorized practices into quintiles based on the number of recruited participants. The distribution was then visualized using a circular bar plot created with the *polarspike* package for Stata, where each bar represents an individual practice [24]. The length of each bar is proportional to the number of recruited participants, allowing for a direct visual comparison of recruitment efforts.

For further analyses, one observation—a large medical center with total staff > 200—was excluded as an extreme outlier.

First, we calculated pairwise Spearman correlation coefficients for the variables of interests and created a heatmap to explore patterns of associations.

To analyse the influence of physician, practice and area-related factors on the number of patients recruited, we then applied a negative binomial regression model with robust standard errors because of an observed overdispersion of the dependent variable [25]. We fit a series of univariable complete case regression models for each independent variable. To verify insights from univariate complete case models, we then conducted a multivariable sensitivity analysis using multiple imputation by chained equations. Missing values in age, gender, practice staff and study workload were imputed using predictive mean matching and (ordered) logistic regression as appropriate, conditioned on the remaining complete covariates. Fifty imputations were generated, and final estimates were pooled following Rubin’s rules [26].

To examine the perceived workload of GPs participating in the trial, a second analysis was carried out using ordered logistic regression in the same manner as described above: a univariable complete case analyses was followed by a multivariable model with multiple imputation of missing data. This analysis was restricted to the questionnaire responders.

Questionnaire response rates ranged from 46 to 63% across quintiles without a clear pattern (*p* = 0.56), suggesting similar engagement levels among responders and non-responders. To further address non-responder bias, a logistic model for study response conditioned on non-missing covariates was used to derive stabilize inverse probability weights, which were applied to multivariate models for adjustment. The significance level was set at α = 0.05.

Stata 15.1 (Stata Corp, College Station, TX, USA) was used for the statistical analysis.

## Results

### Study population: professional, practice and area characteristics

The median age of the study population was 53 years, and 47.6% were male. The majority were specialized in family medicine (77.5%) and held a doctorate degree (75%). About half of them worked in group practices (56.7%), with a median staff of 2 physicians and 4 other healthcare professionals. Most practices were located in urban areas (49.2%) with a median GP coverage level greater than 100% and an intermediate level of deprivation with a median GISD-Score of 0.63 (see Table 1).

**Table 1:** Characteristics of participating GP practices

Sample characteristics	N = 120
GP characteristics	
Age (years), median (IQR)	53 (46–61)
N/A, n (%)	58 (48%)
Gender (male), n (%)	56 (46.7%)
N/A, n (%)	7 (5.8%)
Doctorate degree, n (%)	90 (75%)
Specialty, n (%)	
Family Medicine	93 (77.5%)
Internal Medicine	24 (20%)
No specialty	3 (2.5%)
Additional qualification, n (%)	71 (59.2%)
Recruited participants, median (IQR)	9 (3.5–12.5)
Perceived study workload^1^, median (IQR)	3 (3–3)
N/A	57 (47.5%)
Practice characteristics	
Practice type, n (%)	
Solo practice	52 (43.3%)
Joint practice	68 (56.7%)
Physicians, median (IQR)	2 (1–3)
Practice staff, median (IQR)	4 (3–5.5)
N/A	56 (46.7%)
Area characteristics	
Population size, n (%)	
Rural town (Pop. < 5000)	8 (6.7%)
Small town (Pop. < 20,000)	28 (23.3%)
Medium town (Pop. < 100,000)	25 (20.8%)
Urban area (Pop. > 100,000)	59 (49.2%)
GISD score^2^, median (IQR)	0.63 (0.62–0.74)
GP coverage level^3^ (%), median (IQR)	108.8 (106.8–110.2)

N/A: Not available

^1^Self-reported study workload of participating GPs, assessed on a Likert scale from 1 (low) to 5 (high)

^2^German Index of Social Deprivation (GISD), based on income, education, and employment, ranging from 0 (low/no deprivation) to 1 (maximum deprivation)

^3^GP coverage level, expressed as the ratio of inhabitants to GPs relative to a nationally defined target, accounting for demographic and regional factors

### Recruitment numbers

A total of 1,173 participants were recruited, with 1,030 finally enrolled in the AgeWell.de trial. The lowest quintile (Q1) recruited only 37 participants, accounting for 3.2% of total recruitment, with a mean of 1.5 participants per GP. In contrast, the highest quintile (Q5) recruited 494 participants (42.1%), averaging 22.5 participants per GP. The middle quintiles (Q2–Q4) collectively recruited 642 participants, making up 54.7% of total recruitment (see Table 2).

**Table 2:** Recruited participants per GP practice in quintiles (Q1-Q5)

Quintiles	Number of GPs, *n* (%)	Recruited participants		
		Absolute, *n* (%)	Cumulative, *n* (%)	Mean (SD)
Q1	24 (20,0%)	37 (3,2%)	37 (3,2%)	1.5 (0.5)
Q2	27 (22,5%)	132 (11,3%)	169 (14,4%)	4.9 (1.4)
Q3	28 (23,3%)	269 (22,9%)	438 (37,3%)	9.6 (1.2)
Q4	19 (15,8%)	241 (20,5%)	679 (57,9%)	12.7 (0.9)
Q5	22 (18,3%)	494 (42,1%)	1,173 (100%)	22.5 (9.5)
Total	120 (100%)	1,173 (100%)	1,173 (100%)	9.8 (8.2)

**Fig. 2:**
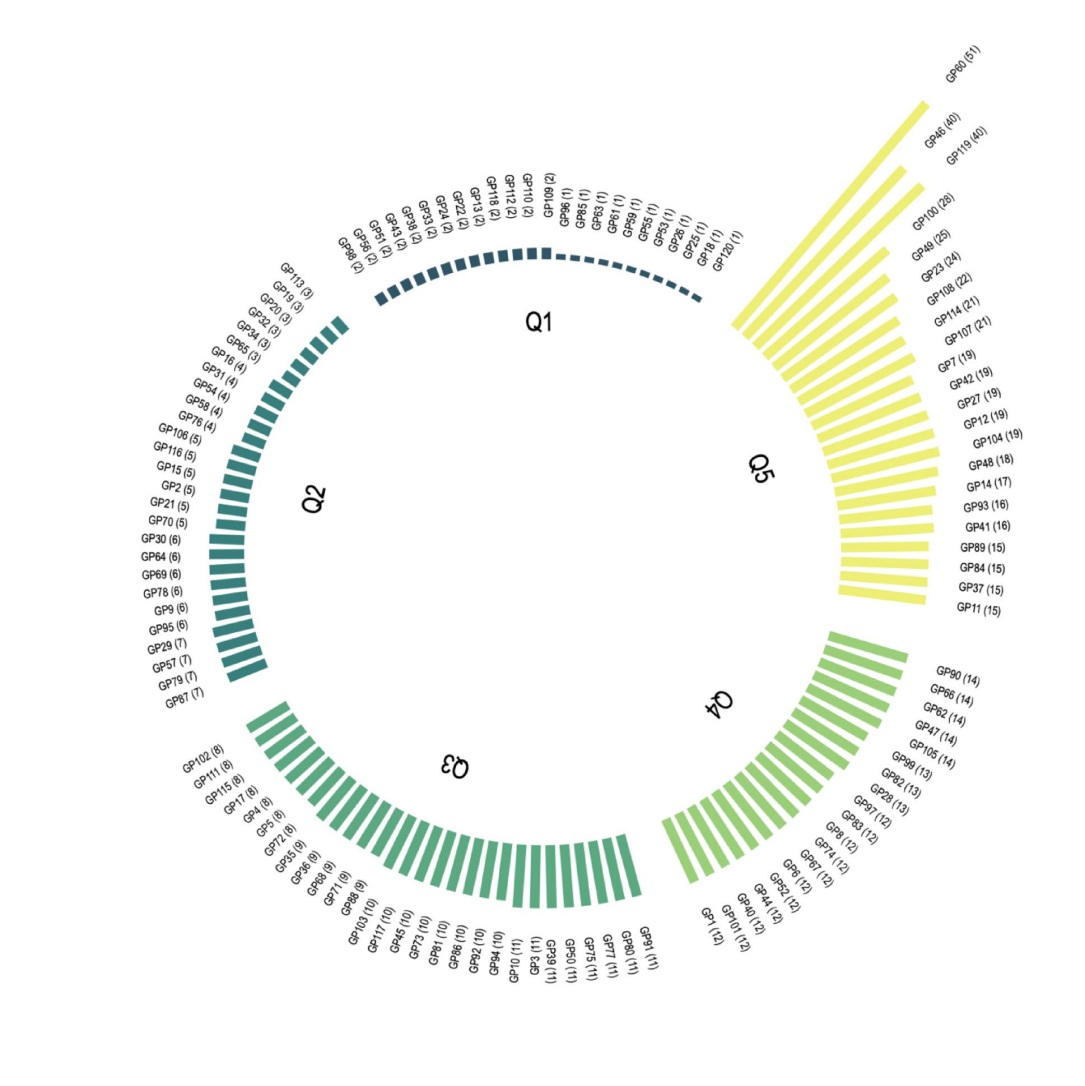
Recruited participants per GP practice. This circular bar plot illustrates participant recruitment across 120 general practices (GPs) in the AgeWell.de trial, with a total of 1,173 recruited participants. Practices are grouped into quintiles (Q1–Q5) based on recruitment numbers, with each bar representing an individual GP office, and its length proportional to the number of recruited participants

Practices in Q5 exceed those in Q1 by a factor of 15 on average, as it can be seen in Fig. 2.

### Correlation patterns

**Fig. 3:**
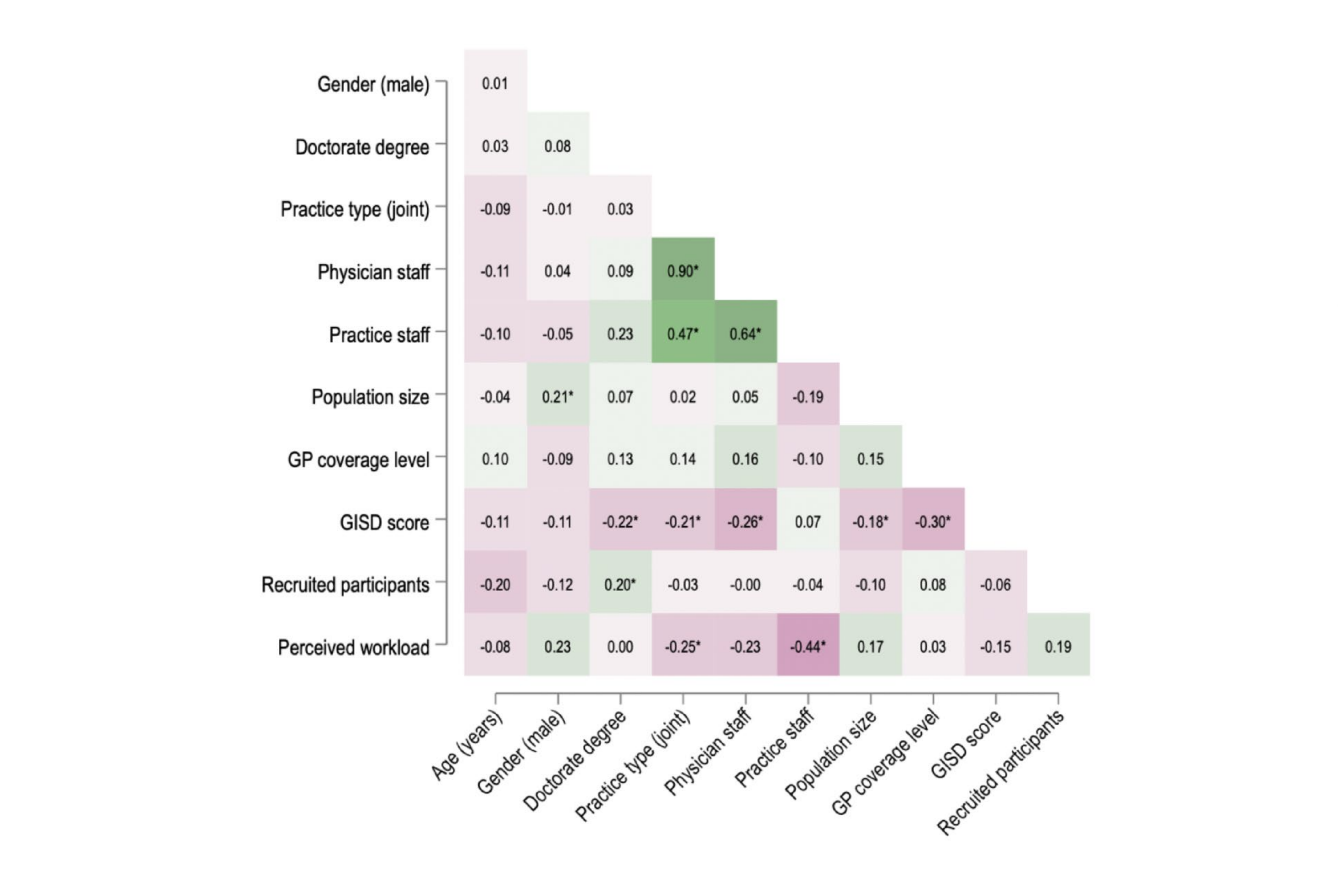
Correlation matrix of key study variables. Heatmap of Spearman’s rho for selected variables, pairwise calculation, n = 119. Positive correlations are shaded in green, and negative correlations in red, with darker hues indicating stronger associations. *p < 0.05

Joint practice structures were strongly linked to more physicians and practice staff (*r*_*s*_ = 0.90 and 0.47, *p* < 0.05). Practices in areas with greater socioeconomic deprivation (higher GISD scores) tended to have fewer physicians (*r*_*s*_ = − 0.26, *p* < 0.05), but not less practice staff. Perceived workload showed a moderate negative correlation with practice size (*r*_*s*_ = − 0.25 to − 0.44, *p* < 0.05). Recruitment numbers did not exhibit strong correlations with structural factors, while in terms of professional attributes, a doctorate degree was positively associated (*r*_*s*_ = 0.20, *p* < 0.05; see Fig. 3).

### Determinants of participant recruitment and perceived workload

In the univariable models, holding a doctorate degree was significantly associated with a higher number of recruited participants (incidence rate ratio; IRR = 1.57, *p* < 0.01), an effect that remained robust in the multivariable model (IRR = 1.45, *p* < 0.05). Male gender showed a weak negative association with recruitment (IRR = 0.75, *p* < 0.1). Perceived workload was positively associated with recruitment (IRR = 1.30, *p* < 0.1). Higher GP coverage levels and age both showed a weak negative association with recruitment in the univariable analysis, though this effect was attenuated in the multivariable model. Other structural factors, including practice size, population and socioeconomic deprivation, showed no clear association with recruitment numbers.

Regarding perceived workload, univariable analysis indicated that joint practice structures and higher practice staff numbers were associated with lower workload, an effect that was partially retained in the multivariable imputed model, where practice staff remained a predictor of lower perceived workload (OR = 0.71, *p* < 0.1), and higher recruitment numbers correlated with greater workload perception (OR = 1.10, *p* < 0.05). Higher GP coverage levels also showed a weak association with increased workload (OR = 1.18, *p* < 0.1) in the multivariable model shown in Table 3.

**Table 3:** Regression models for study recruitment and workload

Variables	Recruited participants^1^		Perceived workload^2^	
	Model 1	Model 2	Model 3	Model 4
	Univariable, complete case	Multivariable, multiple imputation	Univariable, complete case	Multivariable, multiple imputation
Gender (male)	0.76^*^	0.75^*^	1.99	2.70
	[0.57–1.01]	[0.55–1.01]	[0.68–5.84]	[0.63–11.54]
Age (years)	0.98^**^	0.99^*^	0.99	0.98
	[0.95–1.00]	[0.97–1.00]	[0.94–1.04]	[0.92—1.05]
Doctorate degree	1.57^***^	1.45^**^	1.23	0.88
	[1.14–2.17]	[1.03–2.04]	[0.37–4.05]	[0.21–3.78]
Practice type (joint)	0.90	0.83	0.38^*^	1.14
	[0.66–1.23]	[0.60–1.14]	[0.14–1.05]	[0.19–6.95]
Physician staff size	1.03	1.04	0.75^**^	0.92
	[0.96–1.10]	[0.95–1.13]	[0.57–1.00]	[0.53–1.60]
Practice staff size	1.02	1.02	0.60^***^	0.71^*^
	[0.97–1.07]	[0.95–1.09]	[0.42–0.83]	[0.47–1.07]
Population size	0.91	0.94	1.56^*^	1.50
	[0.80–1.05]	[0.82–1.08]	[0.94–2.60]	[0.79–2.84]
GISD score^a^	1.05	0.88	0.12	0.09
	[0.44–2.52]	[0.27–2.87]	[0.01–2.05]	[0.00–36.86]
GP coverage level	0.98^*^	0.98	1.09	1.18^*^
	[0.96–1.00]	[0.96–1.00]	[0.92–1.29]	[1.00–1.39]
Perceived workload	1.22^*^	1.30^*^	–	–
	[0.98–1.52]	[0.98–1.73]	–	–
Recruited participants	–	–	1.03	1.10^**^
	–	–	[0.98–1.08]	[1.00–1.21]
Observations	63–119	119	33–63	66

^1^Negative binomial regression models, reported as incident rate ratios (IRR)

^2^Ordered logistic regression models, reported as odds ratios (OR), restricted to questionnaire responders, with stabilized inverse probability weights (IPWs) applied for non-responder adjustment

^a^German Index of Socioeconomic Deprivation

95% confidence intervals in brackets. ^***^p < 0.01, ^**^ *p* <  0.05, ^*^p < 0.1

## Discussion

### Summary of findings

This study provides insight into what drives GP participation in clinical research, showing that both personal motivation and structural support are important. Scientific training was linked to higher recruitment, while larger practice teams helped reduce perceived workload. However, recruitment was unevenly distributed, with a small group of GPs contributing most and reporting higher workload, raising concerns about sustainability. These findings highlight the need for balanced strategies that encourage participation while preventing overburdening.

### Interpretation of the main results

Since only GPs were invited for study participation, who already had any research or teaching experience with the regional study centers, it can be assumed that they had an higher motivation, increased opportunities, and capabilities for successful patient recruitment than the average GP practice would have.

Practices in more deprived areas tended to have fewer physicians, indicating potential resource constraints that could hinder recruitment efforts. Interestingly, workload perception showed a negative correlation with practice staff, supporting the notion that a larger team can distribute study-related tasks more effectively. However, recruitment numbers did not show strong correlations with structural or regional factors, pointing to individual GP motivation as a primary driver of engagement.

Regression models further underscored the dual nature of research participation, where personal factors drive recruitment, and structural factors influence workload perception. Holding a doctorate degree was the strongest predictor of higher recruitment numbers, emphasizing that scientific training and familiarity with research may foster engagement. Importantly, perceived workload was positively associated with recruitment, suggesting that those who recruited more also felt a greater burden.

Higher GP coverage levels showed weak and inconsistent associations with both lower recruitment and increased workload, suggesting that greater physician density does not necessarily alleviate individual burden.

### Comparison with existing literature

Time constraints, workload, administrative burdens and limited resources are key barriers to GP research participation [10, 27, 28]. In our study, larger practice teams were associated with lower perceived workload as well. Similarly, GPs with research training (doctorate degree) recruited significantly more patients, consistent with evidence that research-experienced physicians have a broader range of motivations for participation [29, 30]. Given that lack of time and research training are frequent barriers [31–33], reducing administrative burdens and offering targeted research training could enhance GP engagement, particularly in smaller or resource-limited practices.

### Implications for research participation strategies

Given the disproportionate recruitment contributions, targeted strategies are needed to support high performers while encouraging broader participation. Low-recruiting GPs may benefit from targeted engagement efforts, such as training, mentorship, or financial incentives. GP teams pronounced professional development and further training of practice staff as promising drivers to improve the motivation and capability of GP teams in terms of patient recruitment and the conduct of clinical trials [34, 35]. Offering regular qualification levels and professional mentoring especially for non-doctoral GPs will guarantee a sustainable implementation of clinical research and the corresponding PBNRs [36]. A monetary incentive does not appear to be superior to a training incentive when it comes to obtaining consent to participate in a study. However, it could offer added value for recruitment success and successful study completion. [37] Meanwhile, high-recruiting GPs reported greater workload, underscoring the need for administrative support, workload compensation, or additional staffing to sustain participation. Given the strong link between scientific training and recruitment performance, PBRNs could help to identify and equip potential high recruiters. Larger practice teams mitigated perceived burden, suggesting that integrating research tasks into routine workflows or hiring research assistants could enhance participation [38, 39]. Lastly, the association between higher GP coverage and increased workload indicates that simply increasing physician density does not reduce burden, emphasizing the need for coordinated infrastructures to facilitate participation in primary care research. Protected research time, mentoring, funding access, and multi-level stakeholder engagement can help address key barriers (i.e., lack of training, experiences and resources to conduct clinical research) [3, 40–43]. Effective planning should involve all stakeholders from study development to execution while considering the entire served population as potential participants [3]. Implementing these strategies requires political commitment and investment in staffing, training, digital infrastructure, and process evaluations [40, 41, 43]. Finally, trustful GP-patient relationships are key to successful recruitment, as they enhance confidence in research participation, particularly in primary care [1, 44, 45]. A strong usual source of care is linked to greater trust in the health system, reinforcing both patient engagement in research and overall healthcare satisfaction [46]. Sustained funding is essential to maintaining these efforts, creating a research-friendly culture that enhances recruitment and advances patient care [4, 42, 47, 48].

Given the complex factors influencing GP research engagement, targeted support is essential to fostering a research-friendly environment [3, 40, 48]. PBRNs present a promising solution to many of the challenges encountered in primary care research by providing structured collaboration, knowledge-sharing, and logistical support [34, 35, 49–52]. Additionally, professional satisfaction, intellectual engagement, and improved patient care motivate GPs to contribute to research, enhancing both recruitment and evidence-based practice [29, 35, 53].

### Strengths and limitations

This study provides one of the first analyses of GP engagement in a large, multicenter primary care trial, linking survey data with public and state-issued records for a comprehensive assessment. Robust statistical methods, including multiple imputation and IPW adjustments, enhanced the reliability of findings. However, the workload analysis was limited to the 55% of GPs who responded to the questionnaire, introducing potential non-response bias despite inverse probability weighting adjustments. The lack of qualitative data on GP motivations and the composition and roles of practice teams prevents deeper insight into recruitment drivers beyond observed associations. Therefore, qualitative analysis will be considered in upcoming process evaluations of clinical trials. Additionally, some GP and practice characteristics were sourced from public databases, which may contain inaccuracies. These data should be validated using additional data sources and might be collected directly in the questionnaire. Generalizability is also limited, as findings stem from a dementia prevention trial and may not fully translate to other study settings. However, most findings might be viewed independently of the clinical question.

## Conclusion

This study demonstrates that both professional and structural factors influence GP participation in research, with scientific training and staff support playing key roles in recruitment performance and workload perception. The uneven distribution of recruitment efforts, highlights the need for balanced engagement strategies. To sustain research participation in primary care PBRNs are crucial. Future research could adopt mixed-method approaches, integrating qualitative insights with quantitative measures of study engagement to refine strategies for enhancing GP participation in clinical trials.

## Electronic supplementary material

Below is the link to the electronic supplementary material.

Supplementary Material 1

Supplementary Material 2

## Data Availability

AgeWell.de data are available for scientific and quality control purposes upon reasonable request based on a data application procedure with the chair of the trial steering committee: Steffi G. Riedel-Heller MD, Institute of Social Medicine, Occupational Health and Public Health, Philipp Rosenthal Str. 55, 04103 Leipzig, steffi.riedel-heller@medizin.uni-leipzig.de.

## Abbreviations

DRKS: German clinical trials register
GISD: German index of social deprivation
GP: General practitioner
IRR: Incidence rate ratio
IQR: Interquartile range
N/A: Not available
OR: Odds ratio
PBRNs: Practice-based research network
Q1-Q5: Quintiles
RCTs: Randomized controlled trials
RKI: Robert Koch institute
SD: Standard deviation
