# Supplementary material for: Two sides of the same coin: recruitment performance and perceived workload in primary care trials-insights from the AgeWell.de study
Source: BMC Prim Care. 2025 Aug 5;26:243. doi: 10.1186/s12875-025-02948-1 (PMC12326823; doi:10.1186/s12875-025-02948-1)
Supplement: Supplementary file 1 — Supplementary Material 1 [file 12875_2025_2948_MOESM1_ESM.pdf]

AgeWell.de – Process Evaluation Questionnaire for Participating General Practices

Age of the participating GP (in years): _______ **Gender:** ☐ Male ☐ Female

Section 1: Workload

**Please rate the overall workload associated with participating in the AgeWell.de trial:**				
**Very Low**	**Low**	**Neutral**	**High**	**Very High**
☐	☐	☐	☐	☐

**Please rate the specific aspects of the workload in more detail:**					
	**Very Low**	**Low**	**Neutral**	**High**	**Very High**
Effort for screening (e.g., eligibility criteria, CAIDE score)	☐	☐	☐	☐	☐
Effort for obtaining informed consent	☐	☐	☐	☐	☐
Effort responding to patient inquiries during the study	☐	☐	☐	☐	☐
Effort for providing feedback to the study team	☐	☐	☐	☐	☐
