# Supplementary material for: Two sides of the same coin: recruitment performance and perceived workload in primary care trials-insights from the AgeWell.de study
Source: BMC Prim Care. 2025 Aug 5;26:243. doi: 10.1186/s12875-025-02948-1 (PMC12326823; doi:10.1186/s12875-025-02948-1)
Supplement: Supplementary file 2 — Supplementary Material 2 [file 12875_2025_2948_MOESM2_ESM.pdf]

Supplementary file 2: Definition of Variables

Variable	*Definition*	Data Source
Gender (male/female)	*Self-reported gender of one lead GP per practice, who was also the documented recruiting physician for the study.*	Process evaluation questionnaire for participating GPs (supplementary file 1)
Age (years)	*Self-reported age of one lead GP per practice, who was also the documented recruiting physician for the study.*	Process evaluation questionnaire for participating GPs (supplementary file 1)
Doctorate degree (yes/no)	*Self-reported doctorate degree of one lead GP per practice, who was also the documented recruiting physician for the study.*	Publicly accessible GP practice websites
Specialty (family medicine, internal medicine, no specialty)	*Self-reported speciality in medical care of one lead GP per practice, who was also the documented recruiting physician for the study.*	Publicly accessible GP practice websites
Additional qualification (yes/no)	*Self-reported additional qualification of one lead GP per practice, who was also the documented recruiting physician for the study.*	Publicly accessible GP practice websites
Practice type (solo practice/ joint practice)	*Self-reported description of the GP practice.*	Publicly accessible GP practice websites
Physician staff size (number)	*Self-reported description of the GP practice.*	Publicly accessible GP practice websites
Practice staff size (number)	*Self-reported description of the GP practice.*	Publicly accessible GP practice websites
Population size (number)	*Publicly available information concerning the number of inhabitants in the area where the GP practice is located.*	Publicly accessible sources
German Index of Socioeconomic Deprivation (GISD score)	*The German Index of Socioeconomic Deprivation is based on income, education, and employment, ranging from 0 (low/no deprivation) to 1 (maximum deprivation).*	Robert Koch Institute (RKI)
GP coverage level (%)	*The GP coverage level is expressed as the ratio of inhabitants to GPs relative to a nationally defined target, accounting for demographic and regional factors.*	Association of Statutory Health Insurance Physicians
Perceived workload (1-5)	*Self-reported study workload of one lead GP per practice, who was also the documented recruiting physician. Workload was assessed on a five-point Likert scale from 1 (low) to 5 (high).*	Process evaluation questionnaire for participating GPs (supplementary file 1)
Recruited participants (number)	*Number of documented patients per GP practice that could be included in the AgeWell.de trial*	Regional study center
